# Graphene and Its Derivatives as Modulators of Macrophage Polarization in Cutaneous Wound Healing

**DOI:** 10.3390/cells14242001

**Published:** 2025-12-16

**Authors:** Iwona Lasocka, Michał Skibniewski, Iwona Pasternak, Anna Wróblewska, Zuzanna Biernacka, Ewa Skibniewska, Lidia Szulc-Dąbrowska, Marie Hubalek Kalbacova

**Affiliations:** 1 Department of Biology of Animal Environment, Institute of Animal Science, Warsaw University of Life Sciences, 02-786 Warsaw, Poland; iwona_lasocka@sggw.edu.pl (I.L.); ewa_skibniewska@sggw.edu.pl (E.S.); 2 Department of Morphological Sciences, Faculty of Veterinary Medicine, Warsaw University of Life Sciences, 02-776 Warsaw, Poland; michal_skibniewski@sggw.edu.pl; 3 Faculty of Physics, Warsaw University of Technology, 00-662 Warsaw, Poland; iwona.pasternak@pw.edu.pl (I.P.); anna.wroblewska@pw.edu.pl (A.W.); 4 Department of Preclinical Sciences, Institute of Veterinary Medicine, Warsaw University of Life Sciences, 02-786 Warsaw, Poland; zuzanna_biernacka@sggw.edu.pl; 5 Institute of Pathological Physiology, 1st Faculty of Medicine, Charles University, 128 53 Prague, Czech Republic; 6 Faculty of Health Studies, Technical University of Liberec, 460 01 Liberec, Czech Republic

**Keywords:** biomaterial, cytocompatibility, cytotoxicity, plasticity, skin

## Abstract

**Highlights:**

**What are the main findings?**

- The modulation of macrophage polarization using graphene-based materials offers a promising approach to regulating wound healing.
- The effects of graphene-based materials may occur either after their internalization by the macrophages or through direct contact in the form of a scaffold or solid surface.

**What are the implications of the main findings?**

- Characterization of the graphene-based materials is essential to assess their action potential (toxicity or compatibility).
- Proper management of macrophage polarization (M1 or M2) using graphene-based materials could enable the design of novel immunomodulatory materials for implantation.

**Abstract:**

Graphene-based materials (GBMs), owing to their excellent biomedical properties, can significantly advance the development of nano-biodressings. Their unique physicochemical features, such as high surface area, tunable functionalization, antimicrobial activity, and ability to interact with immune cells, suggest that GBMs may influence key biological processes involved in tissue repair, particularly the immune response. Building on this growing evidence, the aim of this review is to demonstrate that GBMs can serve as tools for modulating macrophage polarization as a strategy for promoting wound healing. We present the mechanisms by which GBMs penetrate macrophages and discuss the effects of GBMs, either in suspension or as scaffolds/grounds/substrates, on macrophage polarization. Moreover, we propose mechanisms underlying the actions of different forms of GBMs on macrophage polarization. Nevertheless, a multitude of uncertainties and significant challenges remain. Chief among these are the pronounced heterogeneity of GBM subtypes, the necessity for exhaustive characterization and in-depth analysis, the formulation of robust experimental designs, and the careful selection of models capable of accurately delineating macrophage populations and guiding their polarization toward achieving targeted wound healing outcomes. This review attempts to systematize and clarify these issues.

## 1. Introduction

Graphene-based materials (GBMs), with their excellent physicochemical properties, surface modifications, and cytocompatibility, are promising candidates for application in tissue engineering and regenerative medicine. Understanding interactions of GBMs with the immune system is of considerable relevance for wound healing therapy. It is widely acknowledged that macrophages (MØ) have the capability to both destroy and repair tissue. Both processes determine the homeostasis of the wound bed and proper wound healing [1,2]. Appropriate proportions of individual MØ subpopulations and their duration of action during the wound healing phases are crucial. M1 MØ can kill infectious organisms such as bacteria, viruses, and neoplastic cells and phagocyte them. M2 MØ are then involved in tissue remodeling (debris removal, wound healing induction, and angiogenesis) and immune tolerance [3]. The modulation of MØ polarization toward a pro-inflammatory or reparative phenotype by GBMs is a promising strategy to control wound healing. The wound microenvironment is constantly changing, simultaneously influencing MØ polarization. These changes are driven by purely physical and chemical factors. By mimicking these dynamic cues using GBMs, it is possible to modulate MØ populations and, consequently, the wound healing process. However, there are currently no effective therapies that employ GBMs for this purpose. While the factors promoting polarization toward M1 and M2 MØ are well understood, controlling this polarization through GBMs remains a major challenge. Precise characterization of GBMs is key to determining their action potential and should consist of a description of their production method, number of layers, purity, surface chemistry, surface charge, and, in the case of particles, their size, lateral size, dispersibility, and dose. In the case of GBMs as a solid surface, their surface topography, surface roughness, substrate stiffness, and wettability should be considered. Depending on the physicochemical form of graphene, its effects may be exerted after internalization of GBM particles by the MØ or after direct contact with the GBMs layer in the form of a scaffold or solid surface. These impacts can properly manage MØ cell polarity; they can lead to a breakthrough in tissue regeneration and could lead to a new design of immuno-modulating biomaterials for implantation. However, many unknowns and challenges remain. These include the wide variety of GBM forms, the need for thorough characterization and analysis, appropriate experimental design, and the selection of suitable models for identifying MØ populations and directing their polarization to achieve the desired wound healing outcomes. Therefore, we collected and discussed the latest publications on the effects of graphene and its derivatives on the polarization of MØ, which is an important process in wound healing therapy.

## 2. Graphene and Its Derivatives

Graphene is a two-dimensional lattice of carbon atoms [4]. The purest one is only one atom thick. Graphene without defects is called pristine graphene; however, it is uncommon. Various methods of graphene production cause various defects on its surface and in its structure, so “pristine” is often reserved/restricted for graphene material that is one atom thick, 2D, and has minimal defects. The authors indicate that some of these defects are desirable because graphene, in its less-than-perfect form, has a positive effect on cells and can be used for certain applications [4,5,6]. This positive effect can directly regulate the adhesion, proliferation, and differentiation of cells, which are generally regulated by numerous growth factors, environmental stimuli, culture topographies, extracellular matrix (ECM), cell–material interactions, and cell–cell interconnections. Among these factors, cell-material interaction is a challenging factor for the controlled manipulation of cell behavior. The properties of biomaterials, such as their composition, topography, stiffness, and wettability, have been shown to influence cell behavior, mainly through the stimulation of mechanosensors and spatiotemporal dynamics in cells [7,8].

### 2.1. Methods of Graphene Production

Graphene comes in several forms, including graphene film, powder, solution, and paste; graphene nanoplatelets (with a thickness between 1 and 3 nm and lateral dimensions ranging from 100 nm to 100 µm); graphene derivatives like graphene oxide (GO, which is a compound of carbon, oxygen, and hydrogen); reduced graphene oxide (rGO, which has less oxygen and more carbon than GO); and functionalized graphene, when different elements are added to the surface or edges of the graphene for particular applications [9]. Different methods have been developed and used to prepare various dimensions, shapes, and qualities of graphene [9]. For example, graphene films are synthesized by chemical vapor deposition (CVD). The advantages of this method are presented in the work of Lasocka et al. [10], and it was used in the first cell experiment by Kalbacova et al. in 2010 [5].

GO can be produced from graphite [11] or graphene flakes [12] using different chemical oxidation methods. There are three basic methods: Brodie and Staudenmaier, Hofmann, and Hummer [13]. The most widely used method is the Modified Hummer’s Method, which, compared to the original, involves an improved oxidation level [14]. In general, the preparation of graphene oxide is based on the implantation of hydroxy and epoxy functional groups onto a graphene plane and carboxyl groups at the edges [14].

GO prepared by Hummer’s method is very easily reduced by removing functional groups. This form of the material is known as rGO (often called graphene flakes). The reduction process may be influenced by several factors, including chemical reagents, photocatalytic reduction, laser irradiation, plasma, microwave radiation exposure, temperature, light, electrochemical reduction, and microbial reduction [15]. The most common method based on chemical reagents is the use of hydrazine hydrate, because the process is stable and consistent [16]. However, many types of sulfur-containing compounds are also used as effective reducing agents, e.g., Na_2_S, SO_2_, SOCl_2_, and NaHSO_3_ [17].

The reduction of GO leads to the removal of functional groups and the formation of defects, but it is still a good candidate for applications in photocatalysis, water purification, biomedical cover, and biological membranes [18,19,20,21]. This is due to its high wettability and solubility in water [22]. In addition, rGO has been shown to be nontoxic, biocompatible, and antibacterial, and there are a few studies on its use as a membrane in regenerative medicine and for the cultivation of different eukaryotic cells [18,21]. To obtain high-quality graphene flakes, methods other than chemical reduction of GO can be used, including graphite intercalation, electrochemical exfoliation, sonication, or high-shear rotor-stator mixer use. It has also been shown that coatings containing rGO prevented bacterial adhesion to the surfaces [18]. Among the areas of research that have been studied quite extensively is the use of rGO coatings to investigate porphyrin composites for their ability to remove dyes or contaminants from the environment as well as their antibacterial and antifungal potential [19].

In fact, different applications require different grades of graphene, resulting in the widespread practical implementation of this material.

### 2.2. Methods for Graphene Characterization

At first glance, most characterization techniques for thin films, such as graphene, may seem useless; however, they require closer attention. Characterization techniques that can be implemented in graphene inspection include optical microscopy, Raman spectroscopy, Raman mapping, transmission electron microscopy (TEM), atomic force microscopy (AFM), plasmon exciton coupling spectroscopy, scanning tunneling microscopy (STM), scanning electron microscopy (SEM), and many others [23,24]. To present the most useful techniques from a bio-application point of view, we focused on Raman spectroscopy and SEM. Raman spectroscopy was chosen because it allows verification of the type of material used in a fast and nondestructive manner. SEM is an irreplaceable technique for determining the topographies of these materials.

**Figure 1:**
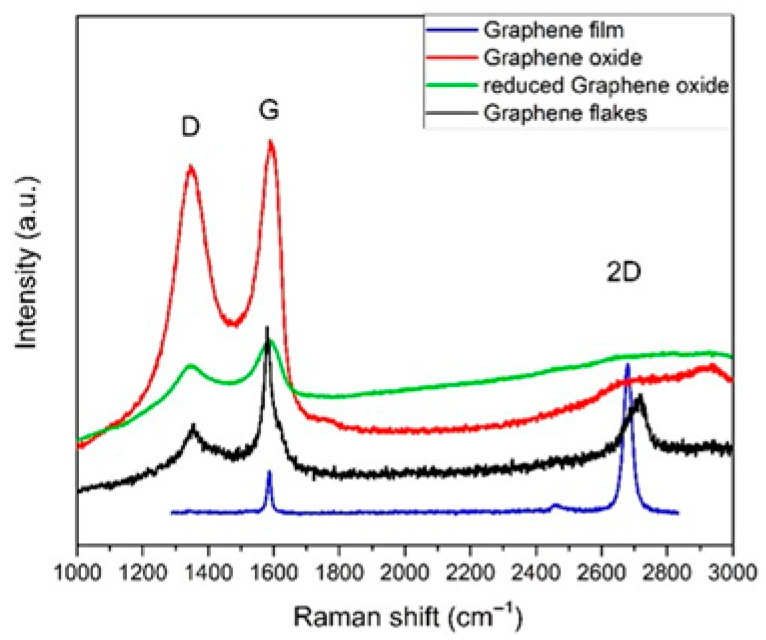
Raman spectra from several carbon nanomaterials exhibiting sp2 bonds. From top to bottom: graphene oxide (red), reduced graphene oxide (green), graphene flakes (black), and graphene films on SiO_2_/Si (blue). Adapted from [10,26,27], authors’ resources.

Raman spectroscopy can be defined as the inelastic scattering of light by matter, from molecules to crystals [25]. The effect is highly sensitive to the physical and chemical properties and the geometric structure of the scattering material. Even small differences in structure lead to significant differences in the observed Raman spectra of the material. Figure 1 compares the Raman spectra of several carbon materials that could be used for MØ polarization.

**Figure 2:**
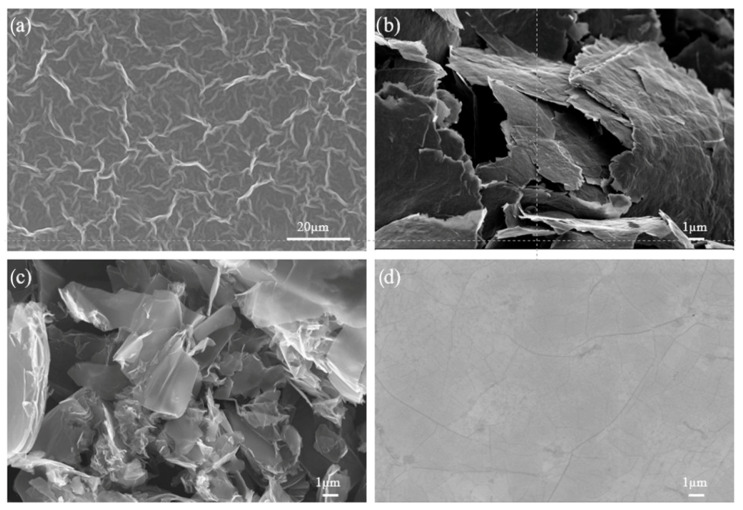
SEM images of (**a**) graphene oxide, (**b**) graphene flakes, (**c**) reduced graphene oxide, and (**d**) graphene films transferred from Cu onto SiO_2_/Si substrate. Adapted from [10,26,27], authors’ resources.

It has been found that different contrasts in SEM images at low acceleration voltages can be attributed to the fact that the intensity of secondary electrons emitted from graphene is affected by different work functions that correspond to the number of graphene layers [28]. SEM imaging also helps to specify the dimensions of graphene features, such as flakes, domains, and bi-tri-layers, or to determine the continuity of the graphene layers. Because of the collection of different types of signals generated as a direct consequence of primary electron and specimen interactions, cracks, folds, and wrinkles, which are crucial for the determination of graphene quality, can be easily distinguished in the SEM images. Figure 2 shows images of GO, rGO, graphene flakes, and graphene films on SiO_2_/Si.

## 3. Wound Healing with Highlights of MØ Function

MØ are the most abundant immune cell type in the skin and play a pivotal role in shaping the immune microenvironment during skin wound healing [2,29,30]. Three terms are inextricably linked to MØ and describe their unique properties: activation, polarization, and plasticity. These processes are discussed below.

MØ are a heterogeneous cell population not only due to their origin but also to their tissue specificity and polarization in response to foreign factors and environmental cues [31,32,33]. Depending on their origin, tissue MØ consist of two classes with phenotypic differences: resident MØ originating from embryonic precursors, and infiltrating MØ derived from monocytes originating in the bone marrow [34,35,36,37]. Both types of MØ (dermal MØ and monocyte-derived MØ) have been found in cutaneous wounds after skin injury [38]. These two primary sources work together to regenerate the injury site, but the content of each type may vary depending upon the stages of wound healing and wound size and depth. In normally healing wounds, the MØ content decreases over time during the repair process [1,39]. MØ also develop distinct phenotypes at different stages of the repair process [31,36]. This process is called MØ polarization and refers to the ability of MØ to change their activation states in response to growth factors (e.g., M-CSF-1 and GM-CSF) and external cues such as cytokines, microbes, infection, and material features such as their structure and morphology [32,40,41,42,43]. The shift from a pro-inflammatory (M1) toward a pro-resolving (M2) profile is triggered by apoptotic cell elimination (efferocytosis), as well as by extracellular vesicles released from apoptotic cells that modify their metabolism [37].

The plasticity of MØ is a very attractive feature for therapeutic targeting, rendering MØ capable of changing their phenotype according to environmental stimuli. The molecular mechanism of M1/M2 MØ polarization remains unclear, but some main signaling pathways may be indicated, including non-receptor tyrosine-protein kinase/signal transducer and activator of transcription (JAK/STAT), interferon regulator (IRF), Notch, and phosphatidylinositol-3-kinase (PI3K/AKT) [44,45,46].

**Figure 3:**
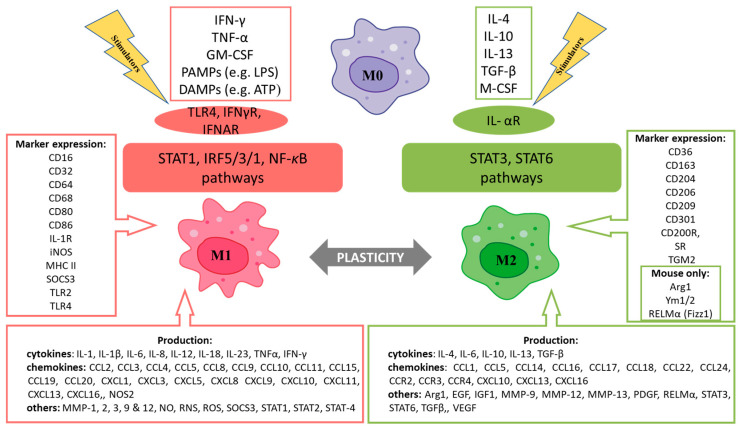
MØ distinct phenotypes (M0, M1, and M2), along with specific receptors, signaling pathways mediating MØ polarization, identifying markers, and secreted factors influencing the wound healing process. Although this graph displays two categories of MØ, in fact a dynamic spectrum of polarization phenotypes occurs during wound healing. Based on: Mooser and Edwards [47], Jablonski et al. [48], Krzyszczyk et al. [36], Aristorena et al. [49], Kim and Nair [32], Yu et al. [50], Peng et al. [51], Saas et al. [37], Liu et al. [52], Hassanshahi et al. [40], Chaintreuil et al. [34], Chen et al. [35], Strizova et al. [53], Zheng et al. [54], and Xue et al. [55]. Abbreviations: Arg1: arginase 1; ATP: adenosine triphosphate; CCL: CC-chemokine ligand; CCR: CC-chemokine receptor; CXCL: CXC-chemokine ligand; CXCR: CXC-chemokine receptor; DAMPs: damage-associated molecular patterns; EGF: epidermal growth factor; GM-CSF: granulocyte–macrophage colony-stimulating factor; IFN-γ: interferon gamma; IFNγR: interferon gamma receptor; IFNAR: interferon-α/β receptor; IGF1: insulin-like growth factor 1; IL-4/13/10: interleukin 4/13/10; IL-αR: interleukin receptors alpha; iNOS: inducible nitric oxide synthase; IRF5/3/1: interferon regulatory factor 5/3/1; LPS: lipopolysaccharide; M-CSF: macrophage colony-stimulating factor; MMP-1/2/3/9/12/13: matrix metalloproteinase; NF-κB: nuclear factor kappa B; NO: nitric oxide; NOS2: nitric oxide synthase 2; PAMPs: pathogen-associated molecular patterns; PDGF: platelet-derived growth factor; RELMα: resistin-like molecule-α (also known as Fizz1); RNS: reactive nitrogen species; ROS: reactive oxygen species; SOCS3: suppressor of cytokine signaling 3; SR: scavenger receptor; STAT1/3/6: signal transducer and activator of transcription 1/3/6; TGF-β: transforming growth factor β; TGM2: transglutaminase 2; TLR4: Toll-like receptor 4; TNF: tumor necrosis factor; TNFα: tumor necrosis factor α; VEGF: vascular endothelial growth factor; Ym1/2: chitinase-like proteins.

In general, phenotypic proportion may dictate the ultimate healing trajectory. Schematically, three main MØ polarization subsets can be distinguished: M0, M1, and M2 (Figure 3).

**Figure 4:**
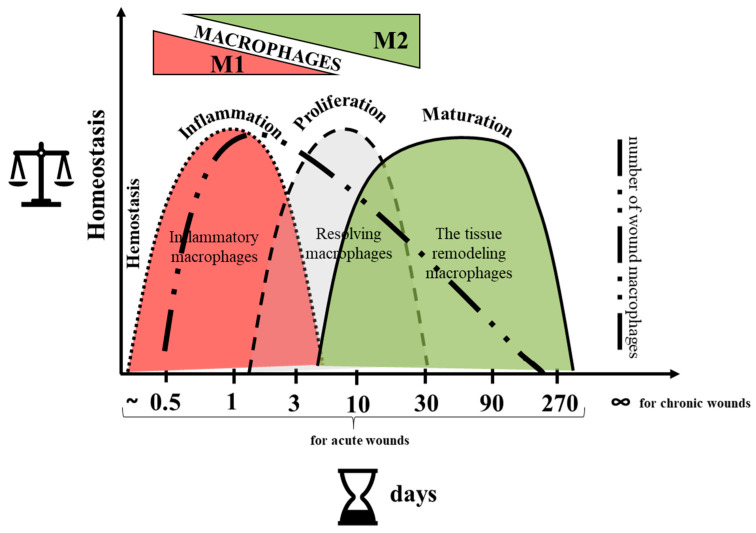
Phases of wound healing with an indication of the types of MØ involved.

Wound healing is a natural, long-term, highly complex, and multi-stage process in which individual phases overlap (Figure 4).

The involvement of MØ in all phases of wound healing indicates their highly dynamic activation diversity and their multidirectional action. These changes are coordinated largely by both tissue-resident MØ and freshly recruited monocytes/macrophages from the blood to ensure the proper course of the skin healing process. Nevertheless, the number of wound MØ gradually decreases to avoid prolonged tissue damage [56] (Figure 4).

Different applications involving skin contact for some GBMs have been proposed; however, they are still in the experimental stage (Table 1) [57,58].

Considering the studies mentioned above, GO is the most widely used, partly due to its antibacterial properties. It shows the best effects when incorporated into other materials, e.g., gels, since GO alone exhibits dose- and time-dependent cytotoxic effects. However, these studies only demonstrate the potential usefulness of GBMs in wound healing and do not document their effect on MØ polarization as a wound healing strategy. The few studies addressing this issue are summarized in Table 2. To properly manage wound healing, the mechanisms of action of GO-based wound dressings should be further explored, and standardized methods for their design need to be established [64]. In the following section, the mechanism of MØ polarization by GO and other GBMs will be proposed.

The classical M1/M2 nomenclature has expanded substantially, as MØ display multiple activation states, including regulatory MØ and distinct M2 subsets (M2a, M2b, M2c, M2d), each defined by specific functions, activation mechanisms, and cytokine profiles [43,47,54,65,66]. These subpopulations range from pro-growth M2a to pro-lysis M2c, with M2b exhibiting mixed pro- and anti-inflammatory properties, and M2d promoting angiogenesis through increased IL-10 and VEGF expression [40,54,67]. Beyond phenotype, MØ identity is shaped by stimulus-specific signatures and metabolic programs, with M1 cells relying on glycolysis and ROS/NO production, while M2 MØ depend on fatty-acid oxidation and glutamine metabolism to support tissue repair [37,68].

Although MØ populations are heterogeneous, it is not easy to follow different types and obtain their exact numbers. Moreover, other factors such as the anatomical location of the wound, the specific region within the wound, the wound environment, and the type of infection have a strong influence on MØ polarization [36]. MØ have the capacity to develop mixed M1/M2 phenotypes, and both these populations often coexist. Moreover, even if there are distinct differences between both polarizations, there are also similarities [69]. Furthermore, the high variability of MØ phenotypes underlies their highly differential involvement in the wound healing process, complicating their prospective use as therapeutic agents.

MØ do not appear as discrete populations in vivo but instead represent a continuum of phenotypes from M1 to M2 that work together to heal the wound [31,43]. Orecchioni et al. [70] indicated that classical and alternative MØ activation in vitro does not match the in vivo M1/M2 polarization. DiPietro et al. [38] also point out that MØ isolated from wounds often do not fall into the categories used in vitro. Many wound MØ express both the M1 and M2 markers. These M1 and M2 markers can be co-expressed by individual cells, highlighting the complexity of polarization states [71]. In vitro studies have attempted to assign specific differentiating factors, surface markers, gene expression, and signaling pathways to MØ populations to control their evolution during wound healing. Plasticity between M1 and M2 MØ varies, and it is suggested that M1 MØ activation has a more restrictive effect on plasticity than M2 MØ activation [32]. This means that the M2 MØ population could be more easily repolarized than M1. It is possible that phagocytosis of senescent neutrophils by MØ could be a trigger for the switch from pro-inflammatory M1 to a reparative M2 phenotype of MØ [38]. Kim and Nair [32] concluded that there is an interdependent, positive feedback loop by which apoptotic cells and Th2 cytokines act together to promote macrophage-mediated wound healing. Moreover, the authors listed other important parameters promoting M2 polarization, such as changes in the ECM structure resulting in cell elongation; increased expression of Arg1, CD206, and Ym1; increased metabolic activity; and a high oxygen consumption rate. Schönborn et al. [72] revealed that mice overexpressing collagen XII exhibit delayed skin wound healing owing to the persistent presence of M1 MØ, resulting in prolonged inflammation.

In summary, during wound healing, the MØ phenotype changes from pro-inflammatory (M1) after injury to regenerative (M2) in later stages. Phenotypic changes over time are a response to microenvironmental signals. Knowing this, we can modulate polarization by introducing the material with a specific nanotopography or nanopatterning, stiffness, size, shape, and layers, for example, GBMs.

## 4. GBMs as an Immunomodulatory Material

The immunomodulatory properties of materials should be a key design factor for new wound healing therapies [36]. If the material possesses the right characteristics, it can be tuned to promote healing. Cutaneous contact is probably one of the most important exposure routes (occupational and/or voluntary) for GBMs [73]. Considering the large variety of GBMs and the lack of their unambiguous effect on innate immune cells, the following is an attempt to summarize the effects of GBMs on MØ. Graphene can modulate MØ behavior in different directions depending on its physical and chemical properties. Examples of such modulations are presented below and show direct contact interactions with graphene derivatives such as scaffold/ground/substrate or dispersed in a suspension.

### 4.1. Modulation of MØ Polarization After Direct Contact with GBMs Suspension

A review of the publications presented below indicates that the response of immune cells to graphene derivatives is crucial and includes MØ plasticity and polarization (pro-inflammatory or reparative phenotype), depending on the GBMs. MØ polarization significantly affects phagocytosis, reactive oxygen species (ROS) production, cytokine release, gene expression, cytoskeleton remodeling, etc. [55]. These changes are described below in the context of the impact of GBMs on MØ.

It seems reasonable to review information about the process of graphene entering MØ, since MØ exhibit significant phagocytic activity and the incorporation of graphene particles into cells can influence their response to functional polarization stimuli [74,75]. For this reason, the first paragraph will focus on the entry of GBMs into MØ cells.

#### 4.1.1. GBMs Entry into MØ

Mendes et al. [76] showed size-dependent uptake of three different nanoscale GO flakes (46, 277, and 453 nm) into undifferentiated human monocyte cells (THP-1) and differentiated MØ. Larger (453 nm) GO flakes (and clusters) were more easily internalized than small individual flakes by both cell types at 10 and 100 μg/mL concentrations. However, this was particularly true for short incubation periods because GO uptake started rapidly within 2 min, and, with longer incubation times, the uptake rate decreased and little difference between samples with various lateral sizes of GO was observed. They also noticed increased uptake with increasing concentrations of GO [76]. Based on TEM studies, the authors revealed that less GO was found inside undifferentiated monocytes than in differentiated MØ [76]. One of the explanations proposed by the authors for this phenomenon is that endocytosis likely occurs at a lower rate in undifferentiated cells. Undifferentiated cells (monocytes) grow in suspension; however, differentiated (MØ) are adherent. Therefore, differentiated cells at the bottom of the Petri dish could be more exposed to larger sedimented flakes/agglomerates of GO. The authors pointed out that GO remained homogeneously dispersed in the medium during the first few minutes, but at the same time, they could not fully exclude the influence of gravity during static cultivation [76]. Based on this research, the authors emphasized that the most important factors determining the rate of absorption of GO on cells are their size and shape. Mendes et al. [76] used GO samples with a thickness of several layers (5–14), whereas Ma et al. [77] used mostly single-layer GO sheets with different lateral sizes (50–350, 350–750, and 750–1300 nm) at a 20 µg/mL concentration searching response in mouse J774.A1 MØ. The uptake of small sheets of GO resulted in an increase (approximately 24–34%) in cellular granularity, as evidenced by the quantitative increase in side scattering, compared to large sheets of GO over the time course of 3, 6, 12, and 24 h. The authors suggested that it may have been related to the inclination of large sheets of GO to associate with the cell membrane [77]. Hoyle et al. [78] showed that small and thin GO sheets are efficiently internalized by immortalized bone-marrow-derived MØ (iBMDMs) prior to LPS priming and localized in phagolysosomal compartments, occupying a large proportion of the cytoplasm, but they do not induce cytotoxicity at any of the concentrations tested, nor when followed by the addition of LPS to the culture medium. TEM images captured by Malanagahalli et al. [79] showed that the internalization of different concentrations of ~3 layers of graphene (~3LG) (3, 10, 30, and 100 µg/mL) into murine bone marrow-derived MØ (mBMDM) occurred mainly by phagocytosis and partly by passive diffusion. Li et al. [80] showed the penetration of the few-layer graphene (FLG) micro-sheets with a range of lateral dimensions (0.5–10 μm) and layer numbers (4–25) into mouse MØ within 5 h of incubation. The authors indicated that the entry was initiated at corners or asperities that were abundant along the irregular edges of the fabricated graphene materials, and this initiated membrane propagation along the extended graphene edge [80]. These authors, in contrast to Ma et al. [77], proposed that this mechanism of spontaneous membrane penetration at the edge allows cellular uptake of even large multilayer sheets of micrometer-scale lateral graphene [80]. Other graphene nanoplatelets (with varied lateral sizes and thicknesses) were observed to be internalized within phagocytic vacuoles of human primary MØ in different amounts and in varying degrees of aggregation [81]. However, no apparent differences in mitochondria, in terms of distortion or damage, or double-stranded autophagosome-like organelles at different concentrations (10, 25, 50, and 100 μg/mL), were observed after 24 h. Artiga et al. [81] found that all tested graphene nanomaterials caused an increase in cytokine IL-8 expression in human primary MØ, and that the response was even more severe for larger graphene nanoplatelets, with IL-8 expression levels similar to those after LPS exposure. The authors proposed a hypothetical mechanism for the biodegradation of carbon nanomaterials (including graphene-based materials), based on the important role of IL-8 released by MØ. IL-8 generally attracts neutrophils, which can then release myeloperoxidase through degranulation. Finally, myeloperoxidase may have the capacity to degrade carbon nanomaterials through their oxidation. It should be noted that the authors did not detect any increase in ROS production after cell administration to any of the tested graphene samples at different concentrations (10 and 50 μg/mL) and incubation for 24 h.

**Figure 5:**
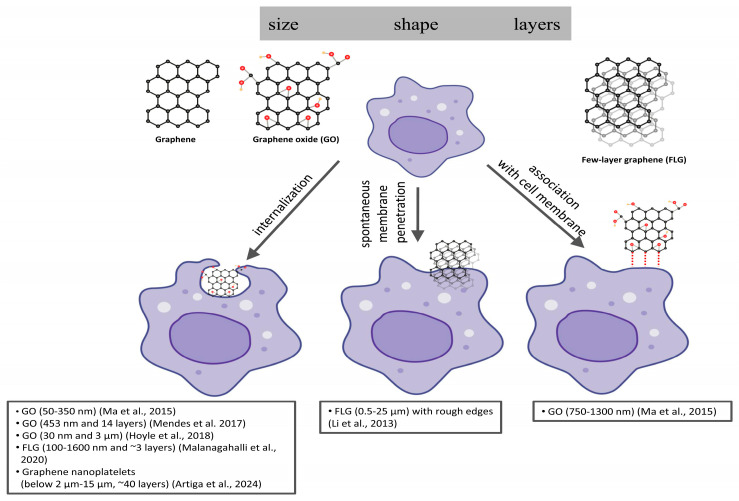
Processes of graphene entering MØ depending on lateral size of GBMs. Based on: Mendes et al. [76], Ma et al. [77], Hoyle et al. [78], Malanagahalli et al. [79], Li et al. [80], Artiga et al. [81]. Detailed explanations are provided in the text.

MØ (macrophages) play a role in the removal of nanomaterial or biodegradation products by phagocytosis [82]. After uptake into MØ, GO was determined to predominantly accumulate in the cytoplasm, causing impairment of the cytoskeleton, as evidenced by cell membrane collapse and reduced ability to phagocytose [83]. On the other hand, fiber-like GBMs promoted human MØ phagocytosis, having no effect on their viability [84]. In turn, Ma et al. [77] presented that smaller GO sheets (50–350 nm in lateral size) were more likely taken up by cells (J774.A1) in comparison to larger (750–1300 nm) GO, which adsorbed more strongly onto the plasma membrane, and their phagocytosis was reduced (Figure 5).

In summary, particle shape, orientation, rigidity, and stiffness play a key role in particle phagocytosis by MØ. Ma et al. suggested that it may be possible to modulate inflammatory responses by controlling the lateral size of GO (as explained above) [77]. The narrower the range of lateral sizes of the GBMs tested, i.e., the more homogeneous they are, and the more precise the conclusions regarding their entry into MØ. The type of nanomaterial itself (GO/nanosheets/nanoplatelets) and its hydrophilicity or hydrophobicity and stability are also important issues.

#### 4.1.2. Impact of GBM Suspension on MØ Polarization

**Figure 6:**
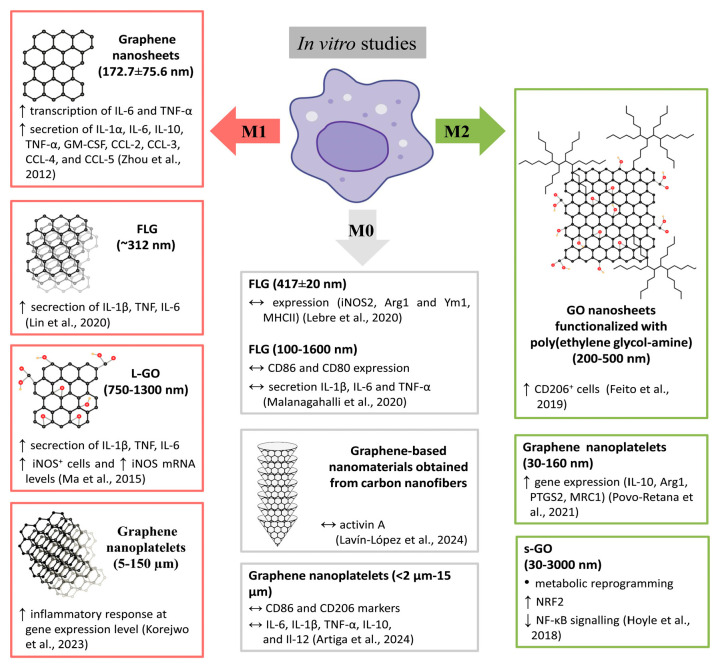
Impact of GBMs suspensions on MØ polarization. Based on: Povo-Retana et al. [75], Ma et al. [77], Hoyle et al. [78], Malanagahalli et al. [79], Artiga et al. [81], Lavín-López et al. [84], Lin et al. [85], Lebre et al. [86], Korejwo et al. [87], Zhou et al. [88], Feito et al. [89]. Detailed explanations are provided in the text.

Povo-Retana et al. [75] studied the interaction of two types of fragmented graphene particles (foam and nanoplatelets) with human MØ: M0 and M0 already differentiated to M1 (LPS + IFNγ + IL1β + TNFα) and M2 (IL4 + IL10 + IL13). They revealed that these two types of graphene particles did not affect cell viability regardless of the polarization conditions. However, the studied forms of graphene enhanced cytoplasmic ROS production only in M0 MØ. In turn, the basal oxygen consumption rate and maximal respiration significantly decreased in M0 and M1 and increased in M2 cells, but only when cells were treated with graphene nanoplatelets. The authors suggested that different graphene forms selectively change the mitochondrial respiratory capacity in a polarization-dependent manner. Of the two forms of graphene studied, nanoplatelets made the main changes in the expression patterns of a series of genes and affected the expression of both pro- and anti-inflammatory responses. However, up-regulation of genes associated with the anti-inflammatory context (e.g., IL10, ARG1, PTGS2, MRC1) suggests the attenuation of the pro-inflammatory response in human MØ [75]. A similar experimental setup was presented in the work by Lin et al. [85], where FLG samples were placed in contact with human M1 and M2 MØ, with a significant increase in the production of inflammatory cytokines (IL-1β, TNF, IL-6) and ROS in M1 MØ compared to control M1 MØ after 24 h of treatment at doses up to 50 µg mL^−1^. No significant decrease in viability was observed in either the M1 or M2 MØ. Moreover, at the highest dose, FLG decreased the expression of the M2 MØ marker CD206 in M2 MØ, but a significant increase in the anti-inflammatory cytokine IL-10 was observed. The authors concluded that the studied doses of FLG failed to evoke an anti-inflammatory response from M2 MØ and that a pro-inflammatory response could be expected as a typical defense mechanism of MØ in contact with foreign particles (Figure 6).

Lebre et al. [86] indicated that FLG was efficiently taken up and retained by mBMDM, without affecting their cellular viability. No signs of specific and certain polarization of these cells toward either phenotype (M1 or M2) were observed (expression of iNOS2, Arg1 for M1 and Ym1 and MHCII for M2 was analyzed). Trained immunity (augmented response of the innate immune cells to a second unrelated stimulus) in mBMDM caused by FLG (with a mean length of 417 ± 20 nm and concentration of 5–80 µg·ml^−1^) was demonstrated, leading to significantly enhanced secretion of IL-6 and TNF-α and decreased secretion of IL-10 following restimulation with LPS. However, the incorporation of graphene into collagen films impaired their training abilities. This indicated the immune-modulatory properties of FLG in long-term studies. In a study by Artiga et al. [81] it was also shown that graphene nanoplatelets did not display any CD86 (marker of M1 MØ) and CD206 (marker of M2 MØ) activation changes, and no significant alterations in IL-6, IL-1β, TNF-α, IL-10, or Il-12 of human primary MØ were found.

GO and graphene nanoplatelets studied by Korejwo et al. [87] affected gene expression in a highly cell type-, time-, dose-, and, unsurprisingly, material-dependent manner. Pronounced changes were observed in the gene regulation involved in inflammatory pathways, mostly for graphene nanoplatelets (5–150 µm), but not for GO (1–50 µm) treated human primary MØ. The authors suspected that the lack of an inflammatory response may have been caused by the large size of the GO studied. Ma et al. [77] found that, compared to smaller (50–750 nm) GO, larger (lateral size between 750 and 1300 nm) GO promoted greater polarization to M1, associated with enhanced production of inflammatory cytokines and recruitment of other immune cells. They also confirmed the ability of GO to polarize mouse MØ toward M1 by examining the induction of Arg1+ cells (representative of M2 MØ) or iNOS+ cells (representative of M1 MØ) by flow cytometry. No significant induction of Arg1+ cells was demonstrated, in contrast to the 31-fold increase in iNOS+ cells for large GO sheets. Moreover, iNOS mRNA levels were significantly elevated—by more than 17-fold—upon treatment with large GO sheets [77,88].

Zhou et al. [88] showed that a suspension of graphene nanosheets interacted with murine MØ and elevated the transcription of IL-6 and TNF-α and the secretion of cytokines (IL-1α, IL-6, IL-10, TNF-α, and GM-CSF) and chemokines (CCL-2, CCL-3, CCL-4, and CCL-5). These results indicate that the graphene nanosheets could trigger pro-inflammatory responses. However, the authors also revealed that these graphene-induced factors can further alter the morphology (remodeling actin assembly) and function of naïve (M0) MØ (decreasing their ability to adhere to the extracellular matrix and attenuate their phagocytosis), suggesting that excessive activation of MØ after graphene exposure can be prevented. Lavín-López et al. [84] showed that at low concentrations, graphene-based nanomaterials obtained from carbon nanofibers (GMC) in suspension did not trigger the release of the inflammatory marker activin A by MØ in the pro-inflammatory phenotype after 24 h of GMC treatment (10 and 20 µg/mL). Additionally, no inflammatory effect of ~3LG on mBMDM was observed by Malanagahalli et al. [79]. Treatment with ~3LG did not significantly affect CD86 and CD80 expression, except for a small decrease in the expression of the second marker at the highest dose (100 µg/mL). No significant increase in inflammasome-dependent IL-1β production was observed, and no significant secretion of other pro-inflammatory cytokines, such as IL-6 and TNF-α, was noted. This indicates that the inflammatory response in MØ was not elicited by ~3LG [79].

The balance between M1 and M2 MØ maintains homeostasis in organisms. This balance, with a slight shift toward the M2 reparative phenotype, was confirmed by Feito et al. [89], who examined the uptake of GO nanosheets functionalized with poly(ethylene glycol-amine) by murine peritoneal MØ at a concentration of 40 μg/mL after 24 and 48 h of treatment (Figure 6). The M1 MØ were characterized by the expression of CD80 and iNOS, and the M2 phenotype was characterized by the expression of CD206 and CD163, detected by flow cytometry and confocal microscopy. The authors noticed a significant decrease in CD80+ cells after 24 and 48 h of treatment and a significant increase in CD206+ cells at 24 h, with this effect reversed at 48 h. The authors suggested that M1 and M2 MØ coexist in the same microenvironment and that nanomaterials could modulate the M1/M2 balance at different incubation times. Changes between M1 and M2 were also reflected in the morphology of MØ: M1 (CD80+) showed a more spherical shape than M2 (CD206+) with an elongated shape [89]. Hoyle et al. [78] revealed that GO alone had no overt pro-inflammatory effect on MØ, but after inflammasome activation with LPS and ATP, inhibited IL-1β release from iBMDMs in a dose-dependent manner. The authors indicated that this inhibitory effect of GO is likely due to the activation of nuclear factor erythroid-2-related factor 2 (NRF2), caused by the metabolic reprogramming of the cell rather than a direct effect on the TLR4−NF-κB pathway [78]. The authors also drew attention to certain key elements during the experiments that influenced their conclusions about the interaction of graphene with MØ (other than material properties, the presence of serum, or endotoxin contamination).

Ma et al. [77] also conducted in vivo studies, indicating that GO, especially in large sheets, could induce enhanced inflammatory cytokine (IL-6 and TNF-α) production locally in the abdominal cavity and systemically in mouse blood, and enhance the recruitment of leukocytes. After administration of large sheets of GO in the abdominal cavities of mice, a 12.2-fold increase in iNOS+ cells (lavage from the peritoneal cavity) compared to untreated cells was observed, indicating MØ polarization to the M1 phenotype.

**Figure 7:**
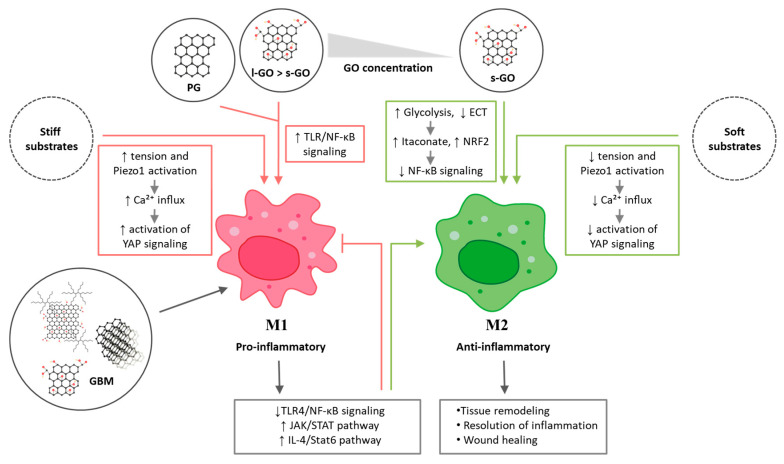
Molecular mechanisms underlying graphene-based regulation of macrophage polarization. Graphene-based materials (GBM) with distinct physicochemical properties and stiffness modulate molecular pathways governing macrophage polarization. Pristine graphene (PG) and large graphene oxide (l-GO), particularly at higher concentrations, enhance TLR/NF-κB signaling pathways, leading to polarization of M1 macrophages. In contrast, small graphene oxide (s-GO) promotes an anti-inflammatory M2 phenotype by reducing NF-κB activity, increasing glycolysis, decreasing electron transport chain (ECT) activity, and elevating itaconate production and NRF2 activation. GBM, particularly GO, can also attenuate pro-inflammatory M1 activation by suppressing TLR4-dependent NF-κB signaling, thereby reducing the expression of cytokines associated with classical activation. At the same time, functionalized GO has been shown to promote the transition toward M2 phenotypes by enhancing IL-4/STAT6 signaling. Stiff substrates increase membrane tension, leading to robust activation of the mechanosensitive ion channel Piezo1. Piezo1-dependent Ca^2+^ influx promotes nuclear translocation of YAP, driving macrophages toward a pro-inflammatory M1 phenotype. On soft substrates, reduced membrane tension limits Piezo1 activation and diminishes Ca^2+^ influx. Consequently, YAP remains predominantly cytoplasmic and inactive, lowering pro-inflammatory signaling and facilitating M2 polarization. PG, pristine graphene; l-GO, large graphene oxide; s-GO, small graphene oxide; GBM, graphene-based material; TLR, Toll-like receptor; NF-κB, nuclear factor kappa-light-chain-enhancer of activated B cells; NRF2, nuclear factor erythroid 2–related factor 2; ECT, electron transport chain; Ca^2+^, calcium ion; YAP, Yes-associated protein; Piezo1, mechanosensitive ion channel Piezo1; JAK, Janus kinase; STAT, signal transducer and activator of transcription; IL-4, interleukin-4.

Many discrepancies have been reported regarding the effect of GBM suspensions on the immune response, which could be attributed to their physiochemical properties, especially the purity, shape, lateral size, thickness, graphene layer arrangements, functional groups, structural defects, and experimental models used. Ensuring the homogeneity and stability of GBMs in the cultivation medium is equally important. However, based on Figure 6, it can be concluded that GO, FLG, and graphene nanosheets alone have a greater tendency to polarize MØ toward the M1 phenotype, whereas GO in the form of a nanocomposite promotes M2 polarization. This is also reflected in the results presented in Table 2, where GO nanocomposites were used and promoted M2 polarization. Furthermore, in Figure 7, the data consistently show that functionalized GO facilitates the transition toward the M2 phenotype [35].

### 4.2. Modulation of MØ Polarization by GBMs as a Scaffold/Ground/Substrate

**Figure 8:**
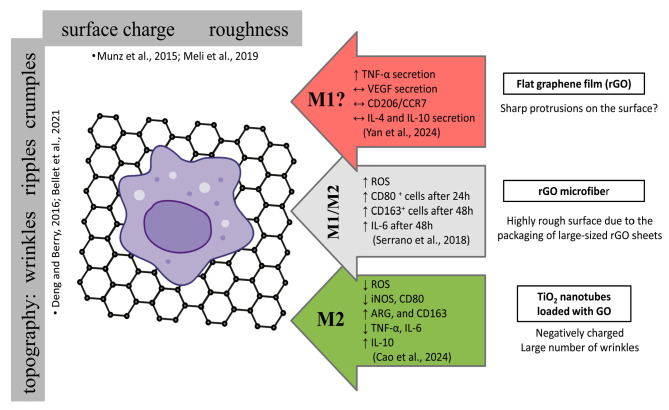
Impact of GBMs as a scaffold/ground/substrate on MØ polarization. Based on: Deng and Berry [90], Bellet et al. [95], Munz et al. [96] Meli et al. [97], Cao et al. [98], Serrano et al. [99], Yan et al. [100]. Detailed explanations are provided in the text.

In contrast to the GBM suspensions, GBMs act as a scaffold/ground/substrate through surface corrugations (wrinkles and ripples) and crumples, which are essential determinants of their biological effects [6,10,90,91,92,93,94,95]. This form of graphene is studied less frequently, probably because of the higher cost of monolayer deposition of graphene on a substrate and the limited possibility of testing in studies. Most often, graphene is deposited on a coverslip on which cells are then seeded [5]. The deposition of a graphene monolayer in wells of a culture dish is not possible unless the graphene is tested in the form of flakes sedimented to the bottom of the wells, but then we cannot talk about a monolayer of graphene. These parameters (graphene monolayer versus few-layer graphene with sharp edges) trigger various mechanisms during cell signal transduction and significantly influence the migration of cells on these substrates. It should be emphasized that graphene monolayers allow only the study of adherent cells. Graphene in the form of a monolayer mimics the substrate onto which it is transferred [5]. As the number of graphene layers increases, the stiffness of the substrate increases along with its hydrophobicity [96]. Meli et al. [97] reviewed the role of biophysical cues in MØ behavior and revealed that the culture of MØ on stiffer materials potentiates inflammatory activation (M1), and grooved or fibrous materials promote alternative activation (M2). A greater M2-like polarization in MØ was also induced by micropatterned posts (spacing between the features had a more significant effect than the size of the features themselves) [97]. They also paid attention to mechanical stimulation modulating wound healing and tissue repair, especially in the skin, because mechanical tension from the contraction of myofibroblasts promotes wound closure. In summary, the mechanosensing of all biophysical cues, including material stiffness, topography, and mechanical forces by MØ, may be exploited therapeutically to treat diseases by modulating the polarization of these cells. Cao et al. [98] revealed that under LPS stimulation, the M1 inflammatory response of mouse MØ (RAW264.7) was reduced, and the M2 polarization of MØ was promoted on TiO_2_ nanotubes loaded with GO. The authors also indicated that the GO coating itself plays an anti-inflammatory (M2) role by reducing ROS production. Moreover, its nanotopography has a favorable regulatory effect on the polarization of MØ toward M2, thereby promoting subsequent tissue healing (Figure 8).

Serrano et al. [99] showed a significant delay in MØ proliferation, without alterations in cell viability and morphology but with a pronounced increase in intracellular ROS content in RAW-264.7 after culture with rGO microfibers. It should be noted that the rGO microfiber surface was very rough, with an RMS value of 399.3 ± 69.6 nm, owing to the packaging of large-sized rGO sheets. The authors revealed that rGO microfibers first (after 24 h) promoted the M1 phenotype of MØ followed by the M2 phenotype (after 48 h) (Figure 8). Moreover, after 48 h of culturing with rGO microfibers, a significant increase in IL-6 levels was observed. The authors suggested that this could have been related to the ROS increase induced by rGO microfibers. However, rGO microfibers maintain the M2/M1 balance–M1 appears first, followed by the M2 phenotype–and this is a key aspect of the immune response during positive tissue repair (Table 2). The authors encourage further investigations of these microfibers as attractive biomaterials that interact with immune cells to support wound healing.

### 4.3. Proposed Mechanisms of GBM-Induced MØ Polarization

Molecular mechanisms governing how GBMs influence MØ polarization involve integrating maternal-specific physicochemical cues with inflammatory and metabolic signaling pathways. Studies indicate that smaller and less oxidized GO preferentially activate the anti-inflammatory transcription factor NRF2 (nuclear factor erythroid 2-related factor 2), triggering metabolic reprogramming characterized by enhanced glycolysis, suppression of the electron transport chain (ETC) activity, and increased accumulation of the metabolite itaconate, which has strong anti-inflammatory properties. These changes dampen pro-inflammatory signaling and promote an M2-like anti-inflammatory phenotype [78]. In contrast, pristine graphene nanosheets activate TLR-dependent NF-κB pathways, leading to robust cytokine production and M1 polarization [88]. It cannot be excluded that a higher stiffness of the graphene material may further amplify the pro-inflammatory response of MØ. It has been found that MØ grown on stiffer substrates tend to adopt a more pro-inflammatory (M1-like) phenotype than those on softer substrates [101]. MØ on stiff substrates experience increased membrane tension, which activates the mechanosensitive channel Piezo1, leading to Ca^2+^ influx and subsequent Yes-associated protein (YAP) activation, which drives a pro-inflammatory M1 transcriptional program. Conversely, soft ECM provides minimal mechanical cues, resulting in weak or absent activation of the Piezo1–YAP signaling axis and thereby favoring MØ polarization toward the M2 phenotype [101]. In line with this concept, the study by Tu et al. [102] demonstrates that increased mechanical cues associated with stiffer substrates drive RAW264.7 MØ toward a more pro-inflammatory state via activation of the RhoA–ROCK/NF-κB signaling pathway. Interestingly, biomaterials can also direct MØ polarization from pro-inflammatory M1 to anti-inflammatory M2, thereby modulating inflammation and tissue repair. This occurs via key signaling pathways, including inhibition of TLR4/NF-κB and JAK/STAT to suppress M1, and activation of IL-4/STAT6 to promote M2 polarization [103] (Figure 7). Therefore, the physicochemical properties and mechanical characteristics of GBMs can be strategically tuned to direct MØ polarization, balancing pro- and anti-inflammatory responses to modulate inflammation and support tissue repair (Figure 7.).

## 5. Challenges in Designing MØ Polarization Studies with GBMs

Guiding MØ polarization is key to faster tissue healing and proper remodeling to achieve a stable wound bed, but it is difficult to select a candidate with the greatest MØ modulatory potential from GBMs (Table 2). One example is an electrical stimulation device that utilizes a graphene film with microgrooves, created by Yan et al. [100], who demonstrated the synergistic effect of microgrooves and electrical stimulation in promoting MØ elongation and polarization to the M2 phenotype. Most studies have documented the beneficial effects of GBMs on skin wound repair in animal models (Table 1). However, to date, few animal studies have specifically investigated the modulation of MØ polarization by GBMs as a potential therapeutic strategy for cutaneous wound healing. In vivo experiments were conducted by Ou et al. [104], who designed a GO-based conductive hydrogel to repair infected diabetic wounds in a rat model of type II diabetes mellitus. The authors used GO as a conductive material, assuming that it could modulate the immune function of the MØ. Moreover, they showed that the conductivity of the designed hydrogel was like that of human skin, and the introduction of GO enhanced not only the mechanical properties of the hydrogel but also its conductivity. The polarization status of MØ was characterized by immunofluorescence staining for CD86/iNOS as an M1 marker and CD206/Arg-1 as an M2 marker. The authors suggested that the GO conductive hydrogel could regulate immune function by enhancing the polarization process to M2 MØ, as determined by the increased number of M2-type MØ in vivo with increased intensity of IL-10–anti-inflammatory cytokines and decreased IL-6, IL-1β, and TNF-α–pro-inflammatory cytokines.

**Table 2:** GBMs as potential modulators of MØ polarization in cutaneous wound healing.

Material	Material Composition and Characterization	Action	Activity	In Vitro/In Vivo	References
Graphene free-standing substrate with 20 µm microgrooves	rGO films micropatterned on the surface of a hydrated PDMS (polydimethylsiloxane) only SEM	Synergic electrical stimulation (DC current 1.5 V/cm) and micropatterning and rGO topography	Cytocompatible, significantly reduced cell spreading area and circularity, MØ M2 phenotypic polarization: ↑ CD206/CCR7 ↓ TNF-α and VEGF secretion ↑ IL-4 and IL-10 secretion Suppression of actin polymerization and PI3K signaling pathway.	THP-1 cells	[100]
rGO microfibers	GO nanosheets SEM, AFM, XPS	Microfibers and rGO nanotopography	MØ M1 phenotypic polarization after 24 h: CD80 +cells have a more spherical shape MØ M2 phenotypic polarization after 48 h: CD163+ cells have a more elongated shape However, no quantitative analysis was performed to confirm these proportions. Additional information is provided in Figure 8.	RAW 264.7 cells	[99]
TiO_2_ nanotubes loaded with GO	GO was electrodeposited onto the surface of anodised TNT (titanium dioxide nanotubes) SEM, AFM, Raman spectra, water contact angle, XRD (X-ray diffraction)	Nanotubes and GO nanotopography	Cells were round or almost round. Promote M2 polarization by downregulating inflammatory and chemokine genes and reducing NADH dehydrogenase activity, NO levels, and ROS production. Additional information is provided in Figure 8.	RAW264.7 cells	[98]
GO-based injectable conductive hydrogel	oxidized hyaluronic acid, N-carboxyethyl chitosan, GO, polymyxin B FITR (Fourier-transform infrared spectroscopy), SEM, conductivity, swelling, stability, structural integrity	Local electrochemical changes (conductive hydrogel)	Cytocompatible, promoting proliferation Spindle-shaped morphology Antibacterial Promote M2 polarization: ↑ number of M2-type MØ ↑ IL-10 ↓ TNF-α Improve the local inflammation, angiogenesis, accelerating the healing of diabetic wounds.	NIH/3T3 fibroblasts *E.coli*, *S. aureus*, Rat Model of Type II Diabetes Mellitus (Sprague–Dawley male rats)	[104]

When considering the potential future applications of GBMs as modulators of MØ polarization, two approaches appear particularly promising: aerosols or foams containing GBMs (nanosheets/nanoplatelets) and plasters composed of a GBM monolayer embedded in parylene C as a substrate. In these contexts, MØ could be directed toward the appropriate phenotype—M1 for pathogen phagocytosis and killing, or M2 for wound healing. However, the mechanisms underlying GBM-induced MØ polarization remain incompletely understood, and therapeutic strategies targeting MØ polarization are still in their infancy.

Certain trends in the effects of GBMs on MØ can nonetheless be observed. GBMs presented as free nanosheets or nanoplatelets tend to induce the M1 phenotype, whereas, when used as a substrate, they favor M2 polarization. Similarly, GBMs incorporated into composites also promote M2 polarization. This summary is highly simplified, as numerous other factors—such as lateral size, number of layers, nanotopography, and surface charge—significantly influence the outcomes (see Figure 6 and Figure 7).

Therefore, it is essential to test a wide variety of GBM forms and identify the most effective configurations for wound treatment. Importantly, research standardization is needed in terms of methods for GBM characterization, MØ identification, and selection of experimental models, both in vitro (including MØ sources) and in vivo, while carefully considering interspecies differences. The key issues related to this topic are described below, whereas the methods for GBM characterization are presented in Chapter 2.

### 5.1. Discrimination of MØ Phenotypic and Sub-Phenotypic States

Identification of MØ phenotypic conversion is challenging due to the lack of a fast and sensitive method to simultaneously detect all classes and subclasses of MØ, especially in vivo, where such a polarization process is highly dynamic and changes over time under the influence of stimuli from the microenvironment [105]. The assessment of MØ polarization depends on the cell origin (primary vs. established cell lines), cell subtype (migratory vs. tissue-resident MØ), and the host (human vs. murine) [106], which will be briefly discussed below in separate subsections.

Currently, the most common techniques used to identify and characterize MØ polarization are based on the assessment of surface and intracellular biomarkers, the expression of specific genes and proteins, the ability to secrete various mediators (such as cytokines and chemokines), and functional analysis (e.g., phagocytosis and production of reactive oxygen species) [43,107]. Nevertheless, it is worth noting that all of these methods have their strengths and limitations. The presence and/or level of biomarkers at the protein level are typically evaluated using flow cytometry [52], Western blot [108], and immunohistochemistry [109]; however, a set of markers, not a single molecule, is required to assess MØ polarization. Such a complex approach provides useful information about effector molecules in different MØ populations, enabling the simultaneous quantification of multiple markers at the single-cell level, which is very important for the analysis of phenotypic cell heterogeneity (flow cytometry) and visualization of marker localization within tissue or cell populations (immunohistochemistry). However, these techniques depend on the quality and specificity of the antibodies, and the phenomenon of cross-reactivity cannot be excluded, which may result in misleading information. Additional limitations include poor phenotypic resolution of similar stimuli, the occurrence of common markers in different polarization states, and the lack of congruence of markers between mice and humans [107].

The analysis of M1/M2 biomarkers may also be performed at the gene expression level using qPCR, high-throughput RNA sequencing (RNA-Seq), and related approaches [96]. Such methods allow the precise measurement of specific polarization markers. RNA-Seq technology not only provides insights into gene expression and other transcriptomic features but also helps identify a greater proportion of differentially expressed genes, particularly those with low expression levels [110]. Therefore, RNA-Seq provides comprehensive transcriptomic profiles at the single-cell level, reveals broader polarization states and plasticity of MØ, and can identify other immune-related pathways [111]. However, RNA-based techniques reflect mRNA levels only at the sampling moment, which may not correlate with real protein activity. The characterization of differences in the expression of mRNAs and proteins simultaneously may be performed by the application of an approach that integrates transcriptomics with proteomics [112]. He et al. [113] combined transcriptomics with global quantitative time-course proteomics and phosphoproteomics to provide a sophisticated multi-layered framework on cellular metabolism, functions, and signaling pathways during polarization initiation in M1 and M2 MØ. However, even such an approach may not reflect the full function of MØ, which are highly plastic and can exhibit features of different phenotypes, and analysis at the population level may mask subpopulations and non-standard MØ phenotypes. This multi-omics approach has both benefits and disadvantages. Multi-omics analysis requires specialized instrumentation and equipment, and the expression of MØ genes/proteins can change dynamically over time and in response to stimuli. Thus, careful interpretation of the results obtained at several time points may be required [111].

MØ polarization can also be assessed based on cytokine/chemokine profiling using ELISA [114] and cytokine arrays [115]. Such techniques provide insights into the MØ functional stage; however, source attribution can be challenging. Furthermore, similar to other techniques that allow functional assessment of MØ (e.g., phagocytosis and nitric oxide production), such functional properties and behaviors may be shared across different phenotypes under certain conditions and are not always feasible in tissue samples [106].

MØ identification and characterization could be improved using novel high-content and high-throughput methods. For example, Geng et al. [107] described a polymer–protein supramolecular assembly as a sensor array that provides an information-rich 5-channel output to rapidly determine MØ polarization phenotypes based on unique fluorescent fingerprints. The sensor is composed of a guanidine-functionalized cationic poly(oxanorborneneimide) polymer and an anionic green fluorescent protein (GFP). When the two entities were assembled into a complex via electrostatic interactions, a Förster resonance energy transfer (FRET) pair was created, and the sensor yielded fluorescent signals in five channels that were quantitatively analyzed using linear discriminant analysis. The authors indicated that their approach offers advantages such as the possibility of simple and rapid determination of MØ plasticity under the influence of subtle environmental changes [107].

Overall, methods for assessing MØ polarization have important advantages, such as precision, high sensitivity, spatial resolution, and the ability to analyze cellular function, as well as limitations related to the lack of existing biomarkers for intermediate MØ polarization stages, different interpretations, high cost, and advanced technical requirements. No single method can comprehensively reflect MØ polarization. In practice, the best approach is to combine several methods to obtain a more complete picture of MØ state and its role in the biological context. The choice of method depends on the research question, available resources, and the sample type. Combining multiple approaches, such as protein markers, gene expression, cytokine profiles, and functional assays, will provide a more accurate and holistic understanding of MØ phenotypes.

### 5.2. MØ Cell Origin (Primary vs. Established Cell Lines)

For in vitro experiments, MØ can be derived from two main sources: primary cells (isolated from tissues or blood) and established cell lines (immortalized cell cultures). As primary cells, peritoneal MØ are commonly used as an in vitro model for studying tissue-resident MØ behavior [116]. In turn, monocytes are isolated from buffy coat-derived peripheral blood mononuclear cells, and CD14+ cells are then purified from this population and differentiated into either M1 or M2 MØ [45]. As a last model, bone marrow-derived MØ are generated by flushing myeloid progenitor cells from the bone marrow of the hind legs of mice and stimulated with either recombinant M-CSF or L-929-cell-conditioned media [116,117]. Immortalized murine MØ RAW264.7 and J774 are the most widely used cell lines for MØ-related experiments and can be obtained from cell banks [116]. The impact of GBMs on them will be discussed below.

In general, primary MØ offer a more physiologically relevant model; however, it is significantly dependent on the donor, whereas the established cell lines are stable and well characterized.

### 5.3. Migratory vs. Tissue-Resident MØ

It is crucial to develop a new generation of biocompatible materials that can mimic dynamic and native tissues and direct host responses to achieve cutaneous regeneration. MØ longevity seems to be an important factor determining the effectiveness of wound healing. Tissue-resident MØ are likely first responders to wounds, are long-lived, and have the capacity for self-renewal [32,36]. They recognize damage-associated molecular patterns (DAMPs) and pathogen-associated molecular patterns (PAMPs) to help fight early infections. Skin MØ can be maintained for an extended period, but with monocyte input [118]. The half-life of dermal MØ was estimated to range from 4 to 6 weeks, while monocytes recruited from the bone marrow die once inflammation is resolved [32]. For that reason, tissue-resident MØ may constitute a better long-term target for wound healing compared to monocyte-derived MØ.

### 5.4. Differences Between Human and Murine Skin and MØ

Skin repair in mice does not fully mirror human healing, but animal models—especially mice—remain essential for studying physiological and pathological mechanisms of tissue repair [36,56]. Despite differences, the main healing phases (Figure 4) are comparable in both species. Their skin consists of three layers: the epidermis (5–10 cell layers in humans vs. 2–3 in mice), dermis (thinner in mice), and hypodermis [36]. Human skin is thicker, firmer, and more tightly attached to underlying tissues, while murine skin is thinner and looser [56]. Wounds also close faster in rodents due to the panniculus carnosus [39]. In mice, healing occurs by contraction and re-epithelialization, whereas in humans mainly through granulation tissue formation and re-epithelialization [36,56]. Mouse skin contains distinct tissue-resident MØ subsets—perineural, perivascular, and perifollicular [119]—which remain difficult to clearly identify in humans due to the lack of unique markers. However, like in mice, human Langerhans cells reside in the basal and suprabasal epidermal layers and function in immune surveillance [120,121]. There are tissue-resident MØ that act similarly to dendritic cells and serve as antigen-presenting cells during inflammation [122,123,124,125].

The general murine MØ markers are F4/80 and CD11b, whereas in humans they are CD14 and CD33 [36]. There are other differences regarding M2 markers: humans lack homologs of mouse Ym1, Fizz1 (RELMα), and arginase-1 [36,43,126,127,128]. Rodents express four RELM proteins, while humans express only two [129]. Moreover, iNOS drives strong NO production in mice, but human MØ do not show such a robust NO response, and the role of iNOS remains unclear [36,43,126,128,130].

Differences also apply to MØ generation for in vitro studies: human MØ are typically derived from peripheral blood monocytes, while murine ones come mainly from bone marrow progenitors [126]. In mice, M-CSF induces MØ formation and GM-CSF drives precursor differentiation into dendritic cells and MØ, whereas in humans, both factors support monocyte survival and differentiation into MØ [126].

In conclusion, some specificities of murine versus human skin and MØ should be considered before using these as models for the evaluation of material interaction with MØ.

## 6. Conclusions

Graphene derivatives are available on the market as single-layer materials, 2–10-layer materials, nanoplatelets, and components of natural or synthetic matrices. All these forms could interact in different ways with the MØ and should be carefully tested before being applied to avoid an unwanted immune response. The possibility of modulating MØ polarization with biomaterials is considered a promising strategy, but the physicochemical properties of GBMs could be responsible for their dual role in the progression or healing of various diseases [1,82]. Precise characterization of graphene is key to determining its action potential (toxicity or compatibility) and should consists of a description of the production method, number of layers, purity, surface chemistry, and surface charge, and in the case of particles, their size, lateral size, dispersibility and dose. In the case of graphene as a solid surface, its surface topography, surface roughness, substrate stiffness, and wettability should be appropriately considered.

Thus far, it is not possible to clearly state which form of graphene and its derivatives influences MØ polarization, and in which direction, whether pro-inflammatory M1 or reparative M2. There are still too few studies dealing with this topic, and too many variables in GBMs can influence MØ plasticity, as was shown in this review. However, our review of published outcomes on this topic indicates that modulating MØ polarization by exerting the influence of GBMs as a suspension or scaffold is possible and could be beneficial in skin wound therapy.

## Figures and Tables

**Table 1:** Dressings containing GBMs for potential clinical use.

Material	Material Composition	Material Properties	Activity	In Vitro	In Vivo	References
GO/Chitosan/Amorphous Calcium Silicate (GC-CS) Aerogel and GO/Chitosan/Calcium Silicate Nanofiber (GC-nfCS) Aerogel	GO, chitosan, calcium silicate aerogels	High liquid absorption capacity (much higher in GC-nfCS but with reduced elasticity and shape retention ability in the wet state)	Good blood compatibility, coagulation, and hemolysis capacity Hemostatic Antibacterial (with photothermal therapy) Wound healing abilities	Rat blood	Rat liver bleeding model BALB/c mice (*S. aureus* bacterial suspension to the wound site)	[59]
Chitosan-rGO-Pluronic F-127 Hydrogel	rGO, chitosan, pluronic F127 (poloxamer 407)	Hydrogel with high mechanical properties, fluid absorption, and conductivity	Antibacterial Enhanced cell migration Enhanced re-epithelialization and vascularization	*E.coli*, *S aureus,* Human dermal fibroblasts	Male Yorkshire pigs	[60]
Alginate foam gel modified by GO	Chitooligosaccharide modified with GO nanocomposite (CG), calcium alginate foam substrate	Foam with high mechanical properties, good water absorption, and stability	Cytocomaptible Antibacterial properties Rapid hemostasis, reducing the inflammatory response and promoting vascular remodeling	NIH/3T3 *S. aureus*	Sprague–Dawley rats	[61]
GO-enhanced chitosan sponge	GO, microcrystalline chitosan, glycerin	Sponge with significant reduction in elasticity after material sterilization; good sorption and absorption properties	Cytocompatible; Antibacterial but not antifungal	L929 fibroblasts *E.coli*, *S aureus*, *C. albicans*		[62]
Nano-silver/GO skin dressing	Ag, GO, gelatin/sodium polyacrylate/kaolin mixture	Hydrogel with good water vapor permeability, high mechanical properties	Infrared bacterial inhibition	*E. coli*		[63]

## Data Availability

All detailed data are available for request in the corresponding authors.
